# Pretibial myxedema treated with intralesional tumescent drug delivery of hyaluronidase, triamcinolone, vitamin B12, and sodium bicarbonate dissolved in a tumescent epinephrine lidocaine solution

**DOI:** 10.1016/j.jdcr.2024.08.044

**Published:** 2024-09-28

**Authors:** Paytra A. Klein, Lauren Voskoboynik, Jeffrey A. Klein

**Affiliations:** a Albany Medical College, Albany, New York; b Department of Internal Medicine, Albany Med Health System, Albany, New York; c Department of Dermatology, University of California Irvine, Irvine, California

**Keywords:** autoimmune disease, epinephrine, glycosaminoglycan, Graves’ disease, hyaluronic acid, hyaluronidase, hyperthyroidism, infiltration, intradermal injection, intralesional, lidocaine, mucin, mucinoses, myxedema, pretibial myxedema, subcutaneous injection, targeted drug delivery, TDD, TEL, TLA, triamcinolone, tumescent delivery, tumescent drug delivery, tumescent epinephrine lidocaine, tumescent epinephrine, tumescent infiltration, tumescent injection, tumescent lidocaine, tumescent local anesthesia

## Introduction

Pretibial myxedema (PTM) is a rare manifestation of Graves’ disease (GD) that involves local inflammation and accumulation of hyaluronic acid (HA)^1^ in subcutaneous and dermal tissue. HA is a glycosaminoglycan that osmotically attracts fluid, causing tissue swelling.^2^ PTM is characterized by bilateral, non-pitting swelling of the anterior shins.^3^ PTM occurs in approximately 0.5% to 4.3% of people with GD, and commonly co-exists with thyroid eye disease.^4^

The term “tumescent” describes a swollen and firm state. A Tumescent Epinephrine Lidocaine (TEL) solution is approximately a 1:11 dilution of a commercial 1% lidocaine with epinephrine 1:100,000 (1% Lido/Epi) solution in normal saline (NS).^5^ This TEL solution is safely and effectively compounded in-office by simply adding 100 mL of 1% Lido/Epi to 1000 mL of NS. TEL is the excipient (carrier solution) for targeted tumescent drug delivery (TDD), which involves targeted, interstitial infiltration of injectable drugs dissolved in a TEL solution.^5^ TDD offers unique pharmacokinetic and pharmacodynamic properties that may provide advantages over intravenous, intramuscular, or per os/oral delivery methods for certain cutaneous targets.^5^

We report the effective treatment of PTM by intralesional TDD of hyaluronidase, triamcinolone, vitamin B12, and sodium bicarbonate dissolved in a TEL solution.

## Case report

**Fig 1:**
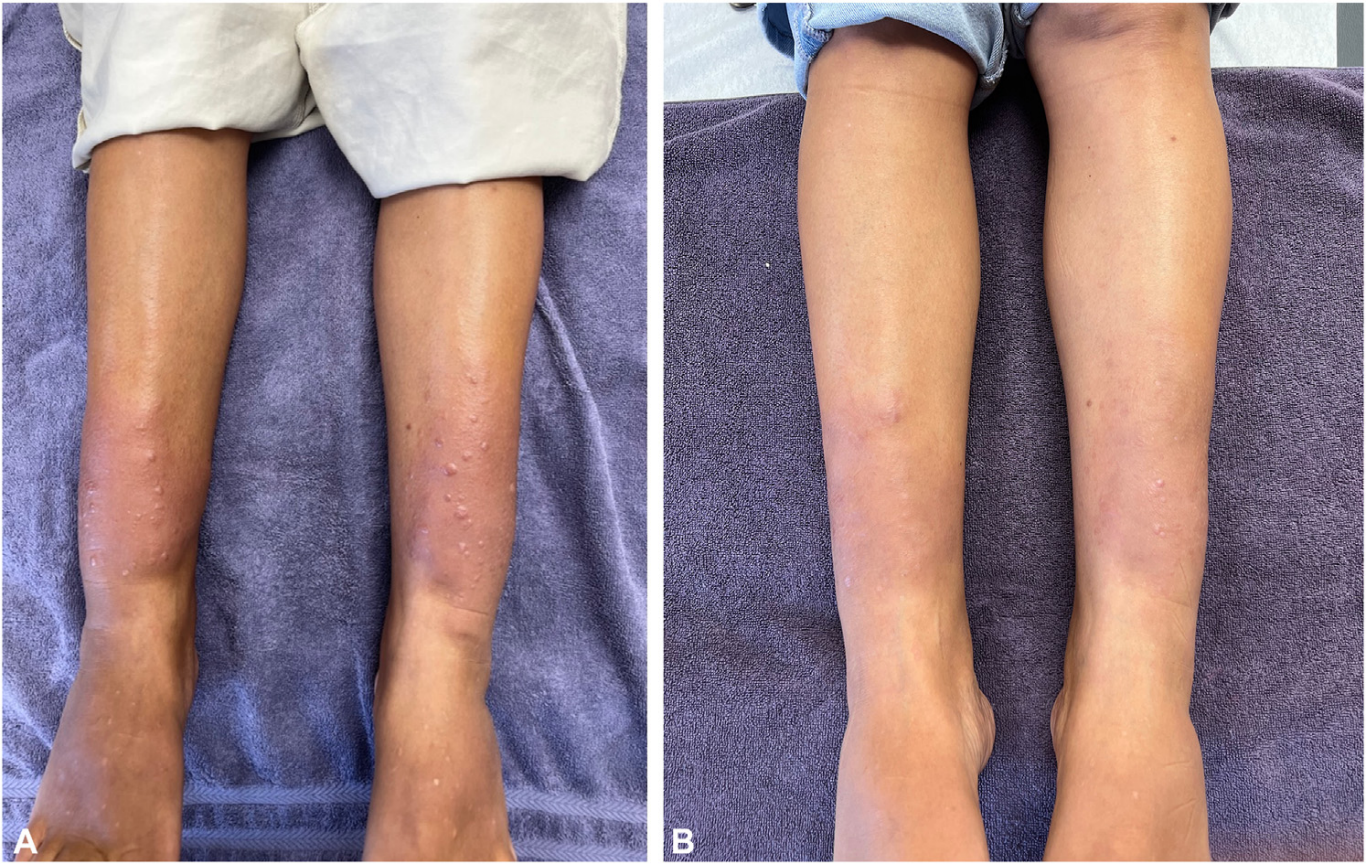
Pretibial myxedema before-and-after treatment. **A,** Pretreatment. **B,** Post-treatment – right leg treated with 450U of hyaluronidase and left leg treated with 300U hyaluronidase, each dissolved in a TEL solution containing triamcinolone, vitamin B12, and sodium bicarbonate. Right and left legs reveal significant and moderate clinical improvement, respectively. *TEL*, Tumescent epinephrine lidocaine.

A 56-year-old female with a history of thyrotoxicosis, thyroidectomy, and GD with exophthalmos presented with a 5-year-history of bilateral erythematous, tender, nonpitting PTM with several mucinous papules (Fig 1, *A*).

We compared the clinical effects of 3 different TDD solutions. These TDD solutions contained either 1 vial (150U; 1.0 mL), 2 vials (300U; 2.0 mL), or 3 vials (450U; 3.0 mL) of hyaluronidase. Each of these 3 TDD solutions also contained 1 ml of triamcinolone (40 mg), 10 ml of 1% lidocaine (100 mg) with epinephrine 0.1 mg, 2 ml of sodium bicarbonate (2mEq), 1 ml B12 (1 mg), and 100 ml of normal saline. Total volumes ranged from 115 ml to 117 ml. The 150U, 300U, and 450U hyaluronidase TDD solutions provided minimal, moderate, and significant clinical improvement, respectively (Fig 1, *A* and *B*). These findings suggest a dose-dependent response of PTM to interstitial hyaluronidase.

**Fig 2:**
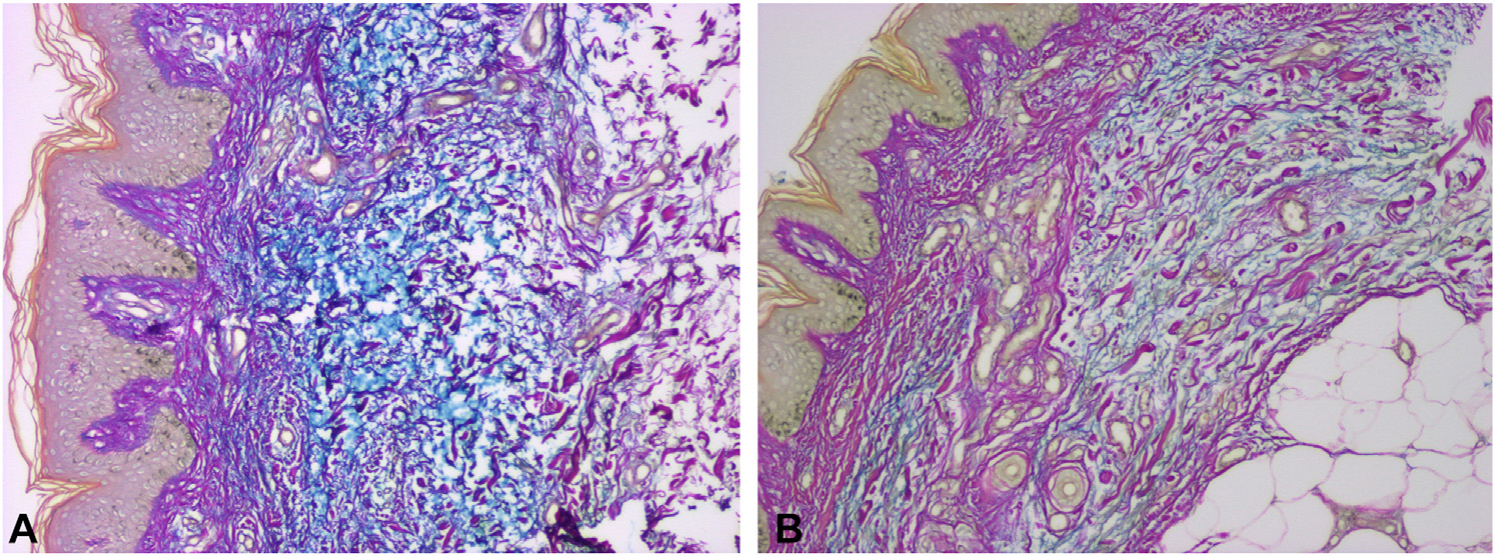
**A** and **B,** Pretibial punch biopsies, stained with colloidal iron, before-and-after treatment with 300U of hyaluronidase dissolved in a TEL solution containing triamcinolone, vitamin B12, and sodium bicarbonate. **A,** Pretreatment punch biopsy of PTM (10×) shows excessive turquois blue-stained mucin throughout the dermis. **B,** Post-treatment punch biopsy (10×) of PTM shows reduction of blue-stained mucin deposits. *PTM*, Pretibial myxedema; *TEL*, tumescent epinephrine lidocaine.

We compared pre- and post-treatment punch biopsies of PTM tissue. A colloidal iron stain revealed extensive dermal mucin in this patient’s pretreatment biopsy, and a reduction of dermal mucin in her post-treatment biopsy (Fig 2, *A* and *B*).

## Discussion

The pathophysiology of PTM in GD is not entirely understood but is thought to mirror that of thyroid-associated ophthalmopathy.^6^ Elevated levels of thyroid stimulating hormone receptor autoantibodies activate thyroid stimulating hormone receptors on fibroblasts in the pretibial and orbital regions.^3^ This stimulates T-cells to secrete inflammatory cytokines that promote glycosaminoglycan (particularly hyaluronic acid) accumulation in the dermis and subcutaneous tissue, causing localized swelling.^3^ HA is a carbohydrate polymer and type of glycosaminoglycan or mucin produced by fibroblasts.

PTM rarely resolves after normal thyroid function is restored.^7^ First line therapy includes topical and/or intralesional corticosteroids with compression therapy.^3^ Second line therapy is oral pentoxifylline.^3^ However, managing PTM remains clinically challenging.

Given that the pathophysiology of PTM involves localized inflammation and interstitial accumulation of HA, intralesional triamcinolone and hyaluronidase (the enzyme that degrades HA) represent a promising treatment for PTM. Recent publications have described effective PTM treatment using intralesional hyaluronidase (150U/mL)^7^ or hyaluronidase (150U/mL) with triamcinolone.^3^

We hypothesize that the pharmacokinetic and pharmacodynamic profile of TDD amplifies the bioavailability of intralesional hyaluronidase and triamcinolone. TDD of hyaluronidase and triamcinolone provides widespread, uniform distribution of these drugs within the targeted tissues, thereby increasing their clinical efficacy.

We report the effective treatment of PTM by intralesional TDD of hyaluronidase, triamcinolone, sodium bicarbonate and vitamin B12 dissolved in a TEL solution.

Hyaluronidase is an enzyme that degrades HA and is considered to be safe.^8^

Hyaluronidase is commonly used off-label to dissolve misplaced or migrated hyaluronic acid filler.^3^^,^^7^ Potential adverse effects of hyaluronidase include allergic reaction, bruising, swelling, and rarely anaphylaxis^8^ or thrombosis.

Triamcinolone is a potent, long-acting anti-inflammatory glucocorticoid that has been found to inhibit glycosaminoglycan synthesis and promote fibroblast degradation.^9^^,^^10^

Epinephrine in a TEL solution produces intense localized capillary vasoconstriction, which the authors hypothesize delays the systemic absorption of every drug in the TEL solution, and thereby amplifies drug bioavailability and local drug effect.^5^

Lidocaine has marked anti-inflammatory effects^5^^,^^11^, ^12^, ^13^ and reduces the localized pain associated with the injection of other drugs dissolved in the TDD solution.^3^^,^^5^ The maximal safe mg/kg dosage of lidocaine in a TEL solution is 21 to 28 mg/kg without liposuction.^5^^,^^14^ Therefore, in a 50 kg person, 21 mg/kg of lidocaine in a TEL solution permits 1,000 ml of TEL.^5^

Sodium bicarbonate neutralizes the pH of TEL, and thus reduces the stinging pain associated with injection of out-of-the-vial commercial lidocaine.

**Fig 3:**
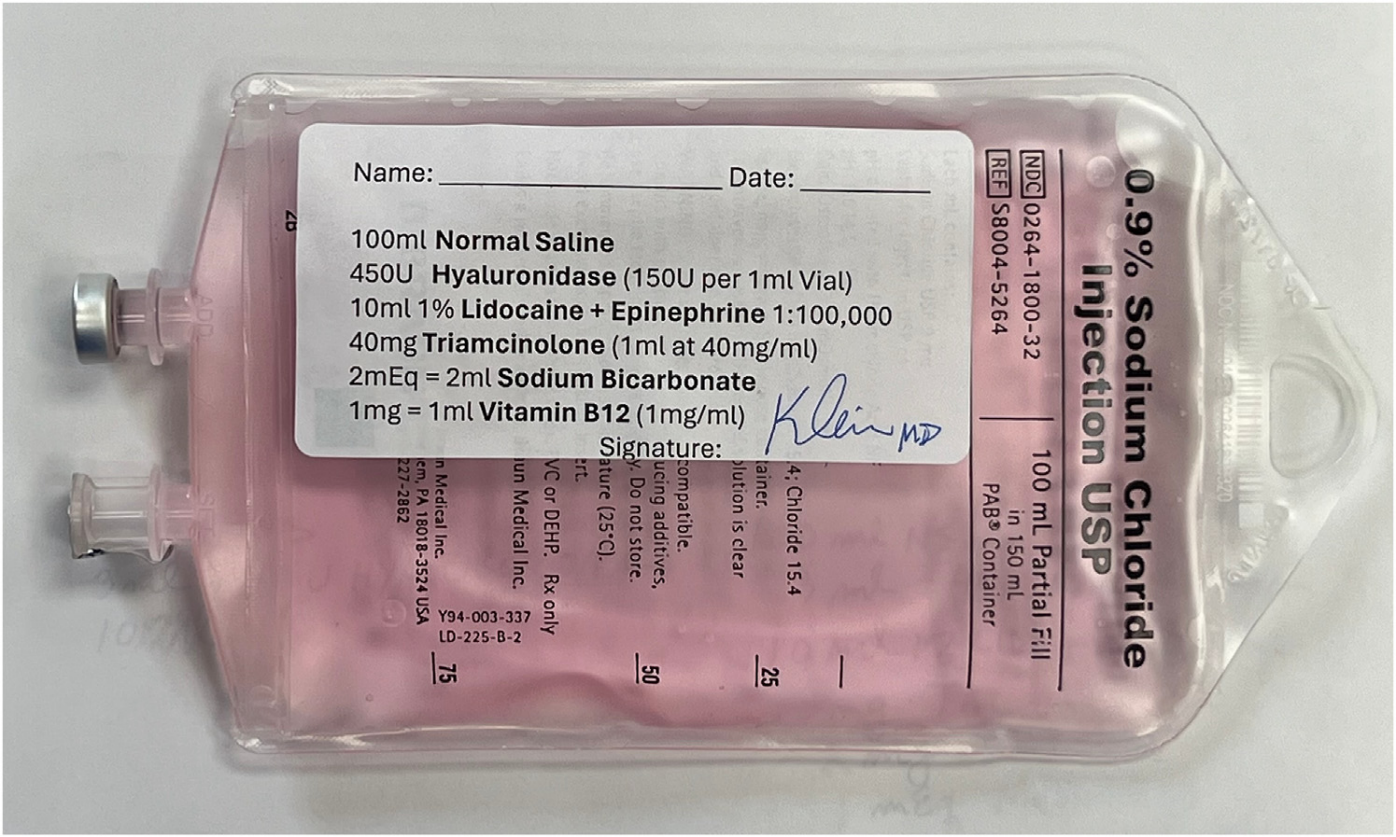
TDD solution containing hyaluronidase, triamcinolone, sodium bicarbonate, vitamin B12, and lidocaine with epinephrine dissolved in 100 ml of normal saline. B12 gives the solution a distinctive *pink* color. *TDD*, Tumescent drug delivery.

Vitamin B12 has anti-inflammatory properties^15^, ^16^, ^17^ and has been found to suppress T-cell cytokine production in vitro.^18^ B12 also gives the solution a distinctive pink color, which provides a visual safety factor that reduces the risk of an unintentional, and potentially fatal, intravenous infusion of a large amount of tumescent lidocaine (Fig 3). The Food and Nutrition Board did not set an upper intake level for vitamin B12 due to its low toxicity risk.^19^ Vitamin B12 is generally deemed safe, even at high doses, because the body does not store excess amounts.^20^ However, side effects of high dose vitamin B12 do exist and include headache, gastrointestinal upset, and fatigue.^21^

**Fig 4:**
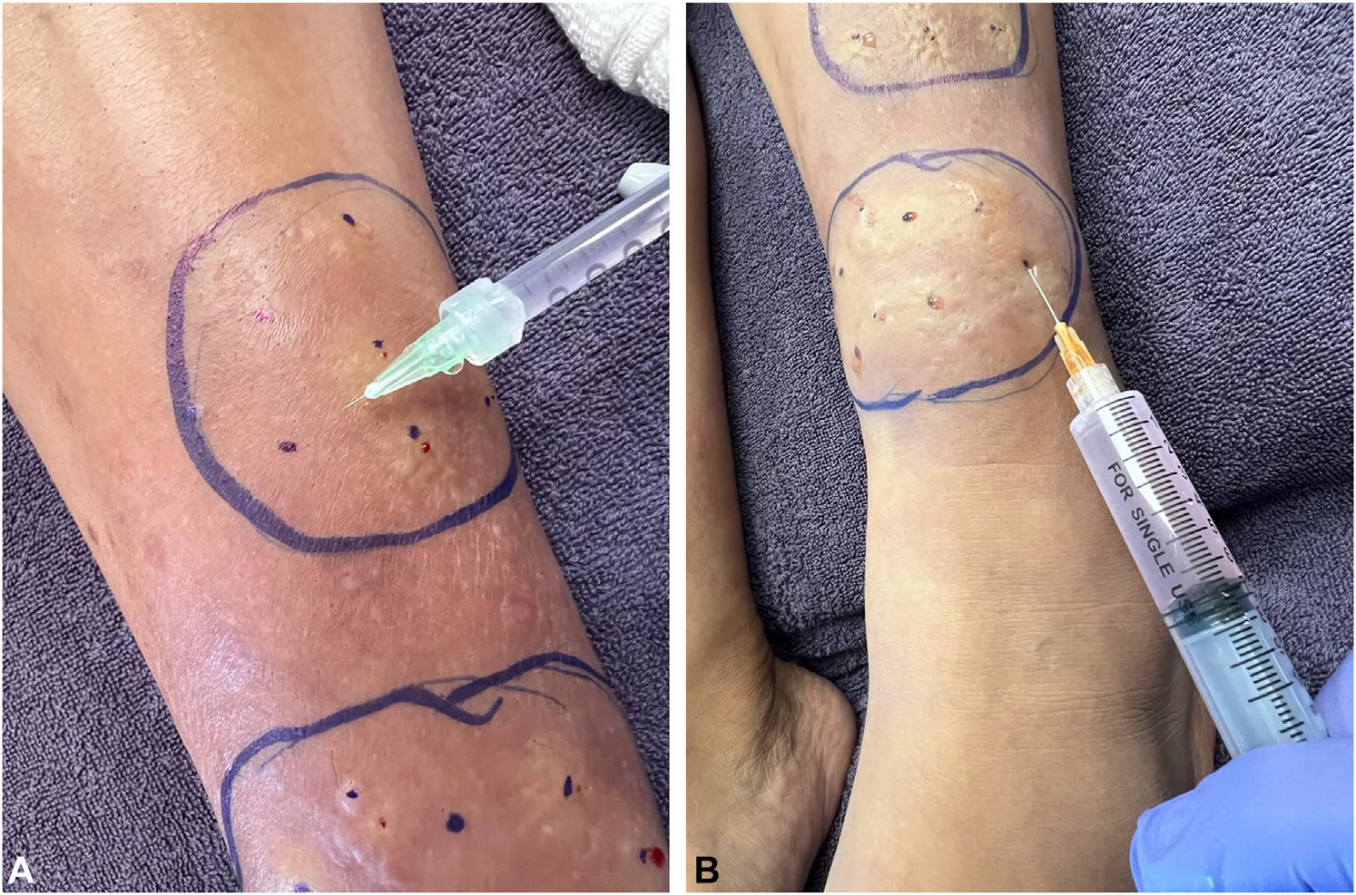
**A** and **B,** Technique for targeted subcutaneous TDD infiltration in PTM. **A,** Shows *encircled* target areas and intended needle insertion sites marked with *dots*. Anesthetic blebs are created using a 32g needle to enable virtually painless subsequent injection of higher volumes of TDD solution (**A**). **B,** Demonstrates percutaneous infiltration using a 25g or 22g needle for administering higher volumes of TDD solution. *PTM*, Pretibial myxedema; *TDD*, tumescent drug delivery.

Painless tumescent infiltration into pretibial tissue is exceptionally challenging. We recommend a slow, gentle, methodical injection technique, using a graduated sequence hypodermic needle sizes (Fig 4). First, use a felt-tipped marker to circumscribe the targeted area and to place dots, spaced 3–5 cm apart, that mark intended needle-insertion sites. Second, with a 32g × 4 mm needle on a 1 ml luer lock syringe containing the TDD solution, inject a pea-sized intradermal anesthetic bleb at each dot. Third, gently insert a 30g × 12.5 mm needle on a 3 ml syringe into each anesthetic bleb to painlessly inject additional TDD solution into mid subcutaneous tissue. Fourth, use a 25g × 25 mm needle, and then a 22 × 25 mm needle, on a 5 ml or 10 ml syringe to painlessly inject sufficient TDD solution into subcutaneous tissue to produce tumescence; this targets hyaluronic acid in the subcutaneous tissue. Lastly, use 32g × 4 mm or 30g × 4 mm needles to painlessly inject the TDD solution directly into the dermis and produce dermal tumescence; this targets intradermal hyaluronic acid.

To the best of our knowledge, this is the first reported case using TDD of triamcinolone and hyaluronidase to effectively treat PTM. We found that PTM has a dose-dependent response to intralesional hyaluronidase in a TEL solution. Since hyaluronidase is not anticipated to affect glycosaminoglycan production by fibroblasts, ongoing intralesional treatments at regular intervals is likely necessary to suppress PTM recurrence.^7^

Future research is needed to investigate potential adjuvant therapies including biologics that target key components in the pathophysiology of PTM, such as rituximab and teprotumumab.^22^^,^^23^ Future studies might explore the use of TDD of triamcinolone and hyaluronidase for the targeted treatment of other cutaneous mucinoses, such as generalized myxedema associated with hypothyroidism, lichen myxedematosus, and scleromyxedema.

## Conflicts of interest

Dr Klein owns HK Surgical, Inc (a surgical supply and equipment company that develops infiltration pumps that can be used for Tumescent Drug Delivery). Dr Klein and Author Klein have patents regarding Tumescent Drug Delivery. Dr Voskoboynik has no conflict of interest to declare.
